# An Adaptive Clustering Approach Based on Minimum Travel Route Planning for Wireless Sensor Networks with a Mobile Sink

**DOI:** 10.3390/s17050964

**Published:** 2017-04-26

**Authors:** Jiqiang Tang, Wu Yang, Lingyun Zhu, Dong Wang, Xin Feng

**Affiliations:** College of Computer Science and Engineering, Chongqing University of Technology, Chongqing 400054, China; yw@cqut.edu.cn (W.Y.); zhulingyun@cqut.edu.cn (L.Z.); wangdong@cqut.edu.cn (D.W.); xfeng@cqut.edu.cn (X.F.)

**Keywords:** hierarchy wireless sensor network with a mobile sink, travel route planning, cluster, multi-hop communication, integer non-linear programming

## Abstract

In recent years, Wireless Sensor Networks with a Mobile Sink (WSN-MS) have been an active research topic due to the widespread use of mobile devices. However, how to get the balance between data delivery latency and energy consumption becomes a key issue of WSN-MS. In this paper, we study the clustering approach by jointly considering the Route planning for mobile sink and Clustering Problem (RCP) for static sensor nodes. We solve the RCP problem by using the minimum travel route clustering approach, which applies the minimum travel route of the mobile sink to guide the clustering process. We formulate the RCP problem as an Integer Non-Linear Programming (INLP) problem to shorten the travel route of the mobile sink under three constraints: the communication hops constraint, the travel route constraint and the loop avoidance constraint. We then propose an Imprecise Induction Algorithm (IIA) based on the property that the solution with a small hop count is more feasible than that with a large hop count. The IIA algorithm includes three processes: initializing travel route planning with a Traveling Salesman Problem (TSP) algorithm, transforming the cluster head to a cluster member and transforming the cluster member to a cluster head. Extensive experimental results show that the IIA algorithm could automatically adjust cluster heads according to the maximum hops parameter and plan a shorter travel route for the mobile sink. Compared with the Shortest Path Tree-based Data-Gathering Algorithm (SPT-DGA), the IIA algorithm has the characteristics of shorter route length, smaller cluster head count and faster convergence rate.

## 1. Introduction

Since it consists of static sensor nodes and mobile sink, the Wireless Sensor Network with a Mobile Sink (WSN-MS) is naturally a tried Wireless Sensor Network (WSN). In the literature [1], WSN is classified into the flat Wireless Sensor Network (fWSN) and the mobile Wireless Sensor Network (mWSN). The fWSN is composed of static sensor nodes and sink nodes, and the data are delivered in a multi-hop manner. Thus, the big problem for fWSN is the hot-spot problem, i.e., the sensor nodes nearby the sink nodes deplete energy quickly. On the contrary, the mWSN has mobile nodes, which can reduce the relay hop count to alleviate the effect of the hot-spot problem. The mWSN is classified into a two-tired Wireless Sensor Network (2-mWSN) and a three-tired Wireless Sensor Network (3-mWSN). In 2-mWSN, static sensor nodes are still the main components laying on the bottom overlay, but mobile devices are introduced as the top overlay. The mobile devices, such as a mobile phone, laptop, Personal Digital Assistant (PDA), mobile robot and drone, can cache and transmit data from static sensor nodes to base station. The literature [2] proposes the new concept of the Wireless Sensor, Actuator and Robot Network (WSARN), which is a kind of 2-mWSN. In 3-mTWSN, static sensor nodes are at the bottom overlay, mobile agents are at the middle overlay, and static access points are at the top overlay. The mobile agents, like animals, vehicles and humans, are responsible for gathering data from static sensor nodes and then forwarding to access points. Data mules in the literature [3], mobile collector (SenCar) in [4,5,6] and Mobility Enabled Wireless Sensor Network Testbed (MOTEL) in [7] are typical 3-mWSN. Because the 2-mWSN has the characteristics of good scalability, avoiding the hot-spot problem and prolonging the lifetime of sensor nodes, in this paper, we are targeting 2-mWSN and propose the corresponding cluster algorithm for sensor nodes and the travel route scheme for the mobile sink.

The key problem of data-gathering in WSN-MS is how to cluster sensor nodes and how to plan the travel route of the mobile sink. Many related works, such as [4,8,9,10,11,12,13,14,15,16,17,18,19], tried to solve these problems. In WSN-MS, sensor nodes are all stationary after being deployed, but mobile sinks have motion ability. Naturally, mobile sinks can move to a position that is close to static sensor nodes to collect data. The direct data-gathering scheme is generated from this thought. In the direct data-gathering scheme, the data-gathering process is divided into data collection cycles. In each cycle, mobile sinks start from the original positions, visit all sensor nodes and finally get back to the original positions. The literature [4,10,20] show that this scheme has many advantages, such as the energy efficiency of sensor nodes and longer network lifetime, etc. However, the travel route of the mobile sink is usually very long, which leads to the significant data delivery latency problem. To avoid these drawbacks, the literature [8,11,12,13] proposes the clustering approach, in which, the WSN is regarded as a kind of hierarchy network, sensor nodes are divided into clusters and mobile sinks move to cluster heads, collecting data. This approach could not only make the travel route of mobile sinks shorter, but also save more energy of the mobile sinks. However, most of the existing works mainly focused on a one or two hop data-collecting scheme, which restricts the route of mobile sinks to be shorter and also limits the energy balance among static sensor nodes. To break these limits, we combine the Route planning problem for mobile sink and Clustering Problem for static sensor nodes (RCP) to make clusters and plan the travel route at the same time.

In this paper, we present the RCP problem by combining the route planning for mobile sink and clustering problem for static sensor nodes. We consider WSN-MS to have static sensor nodes and one mobile sink and make assumptions as follows. First, the static sensor nodes and the mobile sink can estimate their own positions by using related positioning systems or algorithms. We can easily achieve the distances among static sensor nodes. Second, the communication ranges of sensor nodes and the mobile sink are like disks. Given the communication radius, we can calculate the minimum communication hops among static sensor nodes by using shortest path algorithms. Third, we assume the WSN-MS is a kind of 2-mWSN. In the network, static sensor nodes are divided into clusters. In each cluster, there are cluster members and one cluster head. The cluster members send data to the cluster head by multi-hop, and the cluster head stores and forwards the received data to the mobile sink. Based on these assumptions, the data-gathering scheme of WSN-MS could refer to data collection cycles. In each cycle, the data collection process includes three steps. First, the sensor nodes sense the environment and transmit data to the cluster head. Second, the cluster heads store received data and wait for the mobile sink. Third, the mobile sink moves to cluster heads to off-load stored data. In this scheme, the key problem is to plan the travel route for the mobile sink and make clusters for static sensor nodes. In the proposed problem, we set a global control parameter called maximum hops to adjust the balance between the route length and the cluster size. The control parameter is the maximum hops between cluster heads and cluster members.

The contributions of this paper are as follows. First, we formulate the RCP problem as an Integer Non-Linear Programming (INLP). In the formulation, the object is to shorten the route length of the mobile sink where the decision variables include the cluster head vector and the travel route matrix, and the constraints include: maximum communication hops of static sensor nodes, the circular shape travel route constraint of the mobile sink and the loop avoidance constraint of the travel route. From the formulation, we find that the optimal solution with the smaller control parameter is a feasible solution with the larger control parameter. Second, we propose an Imprecise Induction Algorithm (IIA) based on the above property to achieve the approximation solution of the RCP problem. The basic idea of the IIA algorithm is to derive the solution with the larger control parameter from the achieved solution with the smaller control parameter. The process of the IIA algorithm includes two main parts: setting all static sensor nodes as cluster heads, calculating the initial travel route by the Traveling Salesman Problem (TSP) algorithm and iteratively calculating the solution with the larger control parameter from the achieved solution with the smaller control parameter by two kinds of role exchange processes. Third, we conduct extensive experiments that show that the IIA algorithm can solve the RCP problem effectively.

The rest of this paper is organized as follows. Section 2 summarizes the related work. Section 3 introduces the system model, formulates the RCP problem and infers the problem properties. Section 4 proposes the IIA algorithm. Section 5 presents the numerical experiments. Section 6 concludes this paper.

## 2. Related Work

In this section, we go through the related data-gathering approaches in WSN-MS. We roughly divided the data-gathering approaches into three categories from the point of view of sensor clustering: direct data-gathering approach, single-hop data-gathering approach and multi-hop data-gathering approach.

The first category is the direct data-gathering approach, in which the static sensor nodes send data to the mobile sink in a single hop manner, such as in [21,22,23]. In the direct data-gathering approach, static sensor nodes send data to the mobile sink when the mobile sink enters the communication range of static sensor nodes. In [21], Ma et al. focused on the tour planning for a single mobile sink and multiple mobile sinks in WSN-MS. In their study, Mobile data collectors (M-collectors ) start from the static data sink, visit all sensor nodes to gather data and get back to the static sink to upload the data. They found that the network lifetime of WSN-MS is significantly longer than that of static WSN. In [22], Yuan et al. studied the robot routing problem in WSN, in which the robot is regard as the mobile sink. They supposed that the effective ranges of sensor nodes are disks, and the robot must at least reach the boundary to gather data. They proposed an algorithm based on the decomposition of the traveling salesman problem with neighborhoods. For the boundary data-gathering assumption, the route of the robot can be further shortened. In our previous work [23], we study the delivery latency minimization problem in WSN-MS based on the same assumption in [22]. We proposed a substitution heuristic algorithm to achieve an approximate optimal solution and found that the data-delivery latency of WSN-MS is longer than that of static WSN.

The second category is the single-hop data-gathering approach, in which the WSN-MS is organized as clusters, such as [5,6,24,25]. In this approach, the cluster members first send data to the cluster head, then cluster heads store data and wait for the mobile sink, and finally, the mobile sink picks up data from the cluster heads and uploads to the base station. In [24,25], Zhao et al. studied the network cost minimization problem in WSN-MS. They considered that the WSN-MS consists of sensor nodes and anchors. Since anchors are the locations of the parts of sensor nodes, the mobile collector gathers data by visiting each anchor point in a periodic data-gathering tour. They proposed the pricing-based algorithm to determine the data amount and the sojourn time on the anchor points. This work showed that the pricing-based algorithm is better than the cluster-based algorithm, because the aggregate cost is minimized. In [5,6], Zhao et al. studied the dual data uploading in WSN-MS. In their work, the WSN-MS is divided into sensor layer, cluster layer and mobile collector layer. In the sensor layer, they select sensor nodes with higher residual energy as cluster heads. There are two cluster heads in a cluster, and the cluster members communicate with cluster heads within one hop. In the cluster layer, the cluster heads can tune the output power and further adjust the transmission radius. In the mobile collector layer, the mobile collector is equipped with two antennas, and the travel route is designed by the TSP algorithm. This work showed that the dual data uploading approach can consume less energy than the single data uploading approach. All of the works suggest that the single hop data-gathering approach could decease network cost and increase network performance.

The third category is the multi-hop data-gathering approach, which is also organized as a hierarchy network, but cluster members send data to cluster heads by multi-hop. The multi-hop data-gathering approach can be further divided into the hop count-free approach, such as [4,8,9,10,11,12,13,20], and the hop count restricted approach, such as [14,15,16].

The hop count-free approach just focuses on the multi-hop data-gathering mechanism, without considering the tradeoff between energy consumption and data delivery latency. In [20], Wang et al. considered maximizing the network lifetime in a grid-based WSN-MS. In this work, sensor nodes are deployed on a grid, and they can send data to mobile sinks in a multi-hop manner. They proposed a weighted rendezvous planning algorithm, which preferentially designates sensor nodes with the highest weight. In [4], Ma et al. gave the clustering and route planning solutions for connected and disconnected multi-hop WSN-MS. For the connected network, the solution included selecting turning points by reducing the maximum traffic load of the sensor nodes, connecting turning points to form the travel route and obtaining the cluster from the shortest path tree. For the disconnected network, the travel route is divided into inter-cluster circles and inner-cluster paths. These works showed that the multi-hop data-gathering mechanism can prolong network lifetime significantly compared to that in the static WSN.

In [8], Xing et al. tried to find a set of sensor nodes, from which the mobile sinks can pick up the data originating from sources and transport to the base station before the deadlines in a multi-hop manner. In this paper, the routing tree from the sensor nodes to the base station is approximately represented as a geometric tree, and the problem is converted to finding rendezvous points nearby the the geometric tree to satisfy the deadlines. Xing et al. proposed the rendezvous planning algorithm called RP-CP algorithm and the utility-based rendezvous planning algorithm called RP-UG algorithm, respectively. In [9], Salarian et al. proposed a mobile data collection approach based on the rendezvous node in a multi-hop manner. They proposed the weighted rendezvous planning algorithm, which selected the highest workload sensor nodes as the rendezvous nodes. The algorithm also used the classic TSP solver to calculate the tour of the mobile sink. These works showed that the multi-hop data-gathering approach can reduce the data delivery deadline.

In [10], Zhu et al. proposed a tree-cluster-based data-gathering algorithm of the WSN-MS. The algorithm first constructed a weight-based tree and then decomposed the weight-based tree and selected sub-rendezvous points. The algorithm only considers two-hop data communication to limit the energy consumption of sensor nodes. This work shows that the multi-hop data-gathering approach can alleviate the hot-spot problem, balance the load of the whole network and prolong the network lifetime.

In [11,12], furthermore, they studied the network utility problem in the WSN-MS, in which sensor nodes transmit data to the mobile sink in a multi-hop manner. They tried to maximize the data rate of sensor nodes and the flow rate to the mobile sink at certain anchor points. They decomposed the original problem into subproblems and proposed distribution algorithms. In [12], they further introduced the concurrent data uploading mechanism in WSN-MS. Their works show that the multi-hop data-gathering approach can achieve effective network utility under the constraints of network lifetime and data-gathering latency.

In [13], Zhang et al. proposed a hybrid data-gathering approach based on the combination of the hierarchical routing approach and the mobile sink data-gathering approach. In this work, cluster members send data to cluster heads or virtual heads in a multi-hop manner. They selected cluster heads by the node-density-based clustering approach and programmed the travel route of the mobile sink by a low-complexity traveling track planning algorithm. This work showed that the minimum hops in the intra-cluster can save the energy of sensor nodes.

Moreover, the hop count-restricted approach mainly focuses on how to achieve the tradeoff between data delivery latency and energy consumption by adjusting the hop count. In [14], Zhao et al. studied the tradeoff between energy saving and data-gathering latency in WSN-MS by exploring a balance between the relay hop count of sensor nodes and the tour length of the mobile sink. The problem was defined to find a subset of sensor nodes as polling points and the travel route of the mobile sink that connected each sensor in the field to a polling point within given hops, such that the tour length of the mobile sink could be minimized. This paper proposed a centralized algorithm and a distributed algorithm based on the shortest path tree. This paper first proposed that the hops between cluster members to cluster heads should be bounded, so that the balance between energy saving and data-gathering latency could be achieved.

In [15], Bassam et al. tried to find the shortest travel route of the mobile sink to maximize the network lifetime in WSN-MS, where the number of hops between sensor nodes and the mobile sink is bounded. They proposed an energy-aware bounded hop count algorithm, which selected sensor nodes closest to the sink as cluster heads. This work showed that there was a tradeoff between hop count, tour length of the mobile sink and residual energy of the sensor nodes.

In [16], Chowdhury et al. tried to make a better balance between energy consumption and data-gathering latency by adjusting the relationship between the relay hop count for sensor nodes and the route length of the mobile sink. They proposed a data collection points selection algorithm based on the standard shortest path tree. This paper showed that the relay hop count has an impact on the energy consumption of sensor nodes, the data-gathering latency and the route length of the mobile sink.

## 3. System Model and Problem Statement

### 3.1. System Model

We use some assumptions of WSN-MS in [5,6]. The assumptions are as follows.

- Network deployment: The WSN-MS is deployed on a plane randomly. On the plane, there are several static sensor nodes and one mobile sink. The position of the sensor nodes can be achieved by GPS or other locating methods.
- Network architecture: As in [5,6], the WSN-MS is organized as a hierarchical structure, i.e., the static nodes are divided into clusters. A cluster is composed of a cluster head and several cluster members. The cluster member sends data to the cluster head, so the mobile sink could just collect the data of a cluster from the cluster head.
- Network communication: The communication ranges of sensor nodes and the mobile sink are modeled as disks. Two sensor nodes can communicate with each other when their distance is within a given communication radius.

Like [5], we further divide the data collection process into cycles, and in each cycle, the mobile sink starts from the original position, accesses all cluster heads and finally gets back to the original position to prepare for the next cycle. We also assume that the network is organized as clusters, and in each cluster, there are several ordinary sensor nodes and one cluster head. The ordinary sensor nodes, which are also called cluster members, are responsible for sensing and data forwarding. The cluster head is a powerful sensor node, which is mainly responsible for data storage and forward. In our consideration, the cluster member can transmit data to the cluster head in a multi-hop manner. The mobile sink has motion ability; thus in each cycle, the mobile sink accesses all cluster heads one by one. Once clusters are formed and the travel route is planned, the mobile sink can launch data collection cycles. In each cycle, the process is as follows. First, cluster members launch sensing device to obtain data and send the data to the cluster head at the same time. Then, cluster heads store the received data. At last, the mobile sink traverses all cluster heads to collect the stored data.

Based on these assumptions, how to cluster sensor nodes and how to make the travel route for the mobile sink become a critical problem. There are many criteria to cluster sensor nodes and plan the travel route for the mobile sink. However, we just focus on how to select sensor node as the cluster head with the given parameter, i.e., maximum communication hops, to make the travel route of the mobile sink shorter. This problem includes two constraints: communication coverage constraint and route coverage constraint. The communication coverage constraint means that every sensor node should belong to a cluster within given communication hops, so that all sensor nodes can send data to the cluster head. The route coverage constraint means that the travel route of the mobile sink must traverse all cluster heads in each cycle, so that the cluster head can transmit data to the mobile sink.

**Figure 1:**
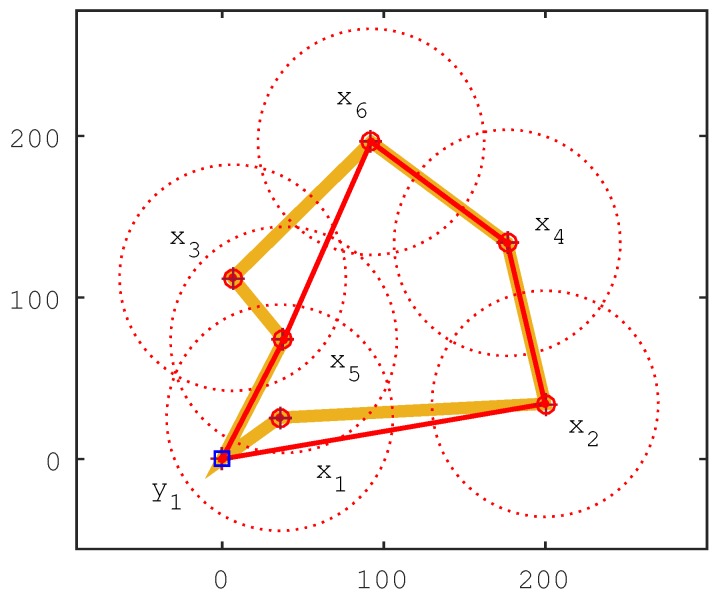
Illustration of the system model.

Figure 1 gives an example of the system model. In this WSN-MS, there are six sensor nodes $x_{1}$, $x_{2}$, $x_{3}$, $x_{4}$, $x_{5}$, $x_{6}$ and one mobile sink $y_{1}$. Every sensor node has the chance to become the cluster head, but sensor nodes $x_{6}$, $x_{4}$ and $x_{2}$ must be the cluster head due to their isolation from others. In the figure, we can see that the sensor nodes $x_{1}$, $x_{3}$ and $x_{5}$ can communicate with each other in a two-hop manner, but the sensor nodes $x_{2}$, $x_{4}$ and $x_{6}$ are zero hops. Thus, the maximum communication hops is two hops, and the minimum communication hops is zero hops. Given the maximum communication hops as zero hops, the shortest travel route is $\left(y_{1},x_{1},x_{2},x_{4},x_{6},x_{3},x_{5},y_{1}\right)$. If the maximum of the communication hops is one hop or two hops, the shortest travel route should be $\left(y_{1},x_{1},x_{2},x_{4},x_{6},x_{5},y_{1}\right)$. The example illustrates the problem that given the maximum communication hops, we can determine the cluster head and program the shortest travel route.

### 3.2. Problem Statement

We present the definition of the RCP problem in WSN-MS as follows.

**Definition 1** (the RCP problem). *Given the set of sensor nodes $X=\{x_{1},x_{2},\ldots ,x_{n}\}$, the position of sensor nodes $\bar{X}=\left\{{\bar{x}}_{1},{\bar{x}}_{2},\ldots ,{\bar{x}}_{n}\right\}$, the maximum hops between cluster heads and cluster members $H_{max}$ and the maximum communication radius $R_{max}$, the RCP problem is defined as an optimization problem to achieve the shortest travel route of mobile sink $y_{1}$ by determining the set of cluster heads H and planning its access sequence ρ.*

First, we define the decision variables $U=\{u_{1},u_{2},\ldots ,u_{n}\}$ and $V=\{v_{11},v_{12},\ldots ,v_{nn}\}$. The variable *U* denotes whether sensor nodes are selected as cluster heads. The elements of *U* is defined as follows.

(1) $$\begin{array}{c} u_{i}=\left\{\begin{array}{cc} 1 & \;\;(x_{i}\in H)\\ 0 & \;\;(x_{i}\notin H) \end{array}\right. \end{array}$$

The variable *V* denotes whether edges are selected as route segments of the shortest travel route, denoted by a matrix as follows.

(2) $$\begin{array}{c} V=\left(\begin{array}{cccc} v_{11} & v_{12} & \cdots & v_{1n}\\ v_{21} & v_{22} & \cdots & v_{2n}\\ \vdots & \vdots & \ddots & \vdots \\ v_{n1} & v_{n2} & \cdots & v_{nn} \end{array}\right) \end{array}$$

The elements of *V* are defined as follows.

(3) $$\begin{array}{c} v_{ij}=\left\{\begin{array}{cc} 1 & \;\;(e_{ij}\in \rho )\\ 0 & \;\;(e_{ij}\notin \rho ) \end{array}\right. \end{array}$$

Then, we give the mathematic expression of the relationships among sensor nodes. Once the WSN-MS is deployed, we can achieve the distance and communication hops among sensor nodes. The distance and the communication hops are denoted by $M_{d}$ and $M_{h}$, respectively.

(4) $$\begin{array}{c} M_{d}=\left(\begin{array}{cccc} d_{11} & d_{12} & \cdots & d_{1n}\\ d_{21} & d_{22} & \cdots & d_{2n}\\ \vdots & \vdots & \ddots & \vdots \\ d_{n1} & d_{n2} & \cdots & d_{nn} \end{array}\right) \end{array}$$

where $d_{ij}=∥{\bar{x}}_{i}-{\bar{x}}_{j}∥$.

(5) $$\begin{array}{c} M_{h}=\left(\begin{array}{cccc} h_{11} & h_{12} & \cdots & h_{1n}\\ h_{21} & h_{22} & \cdots & h_{2n}\\ \vdots & \vdots & \ddots & \vdots \\ h_{n1} & h_{n2} & \cdots & h_{nn} \end{array}\right) \end{array}$$

where $h_{ij}$ is the minimum hops between sensor node $x_{i}$ and sensor node $x_{j}$. If $x_{i}$ cannot reach $x_{j}$, $h_{ij}=\infty$, and if i=j, $h_{ij}=0$. The matrix $M_{h}$ can be derived by classical shortest path algorithms, such as, the Dijkstra algorithm [26] and the Floyd–Warshall algorithm [27].

At last, the RCP problem can be formulated as an INLP problem.

(6) $$\begin{array}{c} RCP:\min f\left(U,V\right)=\sum_{i=0}^{n}\sum_{j=0}^{n}u_{i}u_{j}v_{ij}d_{ij}\\ s.t. \end{array}$$

(7) $$\begin{array}{c} \left(1-u_{i}\right)\min_{j=0}^{n}u_{j}h_{ij}\leq H_{max},\left(i=0,1,\ldots ,n\right) \end{array}$$

(8) $$\begin{array}{c} \sum_{i=0}^{n}u_{i}u_{j}v_{ij}=u_{j},\left(j=0,1,\ldots ,n\right) \end{array}$$

(9) $$\begin{array}{c} \sum_{j=0}^{n}u_{i}u_{j}v_{ij}=u_{i},\left(i=0,1,\ldots ,n\right)\\ u_{i}u_{j}\left(w_{i}-w_{j}+v_{ij}\left(n+1\right)\right)=u_{i}u_{j}n, \end{array}$$

(10) $$\begin{array}{c} \left(i,j=0,1,\ldots ,n-1\right) \end{array}$$

(11) $$\begin{array}{c} v_{ij}\in \left\{0,1\right\} \end{array}$$

(12) $$\begin{array}{c} u_{i}\in \left\{0,1\right\} \end{array}$$

(13) $$\begin{array}{c} w_{i}\in \left\{1,2,\ldots ,n\right\} \end{array}$$

where *U* and *V* are decision variables, $h_{ij}$ is from the matrix $M_{h}$, $H_{max}$ is the maximum communication hops between cluster heads and members and $w_{i}$ is the temporary variable. The explanation of the constraints is as follows.

- Equation (6) is the object function, which is to minimize the length of the travel route traversing all cluster heads.
- Equation (7) is the communication hops’ constraint, which restricts the maximum hops among cluster members and cluster heads.
- Equations (8) and (9) are the travel route constraints. Expression (8) denotes that one cluster head has only one edge entering, and Expression (9) denotes that one cluster head has only one edge leaving.
- Equation (10) is the loop avoidance constraint, which means that there is only one loop on the travel route.
- Equations (11) and (12) set decision variables as binary.
- Equation (13) sets the temporary variable as the sequence number of sensor nodes. Actually, the temporary variable can be a real number.

### 3.3. Problem Property

From the formulation, we can derive the following properties.

**Theorem 1** (Feasible solution). If $\left(U_{k}^{*},V_{k}^{*}\right)$ is the optimal solution under the condition $H_{max}=k$, $\left(U_{k}^{*},V_{k}^{*}\right)$ is a feasible solution under the conditions $H_{max}=k+1$ and $f\left(U_{k}^{*},V_{k}^{*}\right)\geq f\left(U_{k+1}^{*},V_{k+1}^{*}\right)$.

**Proof of** **Theorem 1.** If $\left(U_{k}^{*},V_{k}^{*}\right)$ is the optimal solution when $H_{max}=k$, we can infer that $\left(U_{k}^{*},V_{k}^{*}\right)$ satisfies Constraints (7)–(13). For $\left(H_{max}=k\right)<\left(H_{max}=k+1\right)$, when $H_{max}=k+1$, $\left(U_{k}^{*},V_{k}^{*}\right)$ satisfies Constraint (7). Because $H_{max}$ is not a parameter of Equations (8)–(13), $\left(U_{k}^{*},V_{k}^{*}\right)$ satisfies Constraint (8)–(13), when $H_{max}=k+1$. In total, $\left(U_{k}^{*},V_{k}^{*}\right)$ is a feasible solution when $H_{max}=k+1$.

$\left(U_{k}^{*},V_{k}^{*}\right)$ is just a feasible solution, so it is equal to or greater than the optimal solution, i.e., $f\left(U_{k}^{*},V_{k}^{*}\right)\geq f\left(U_{k+1}^{*},V_{k+1}^{*}\right)$. ☐

**Theorem 2** (*H_max_* = 0). *If $H_{max}=0$, the RCP problem is a TSP problem.*

**Proof of** **Theorem 2.** If $H_{max}=0$, all sensor node are cluster heads. The mobile sink must access all sensor nodes by traversing the transmission area of all sensor nodes; thus, the RCP problem is a TSP problem. ☐

## 4. Imprecise Induction Algorithm

### 4.1. Basic Idea

From the inspiration of Theorem 1, we propose an Imprecise Induction Algorithm (IIA). Theorem 1 tells us that the optimal solution with small maximum hops is a feasible solution with large maximum hops in the RCP problem. Therefore, we can solve the RCP problem from the smallest maximum hops, i.e., $H_{max}=0$, and then, we can derive the solution with $H_{max}=1$, $H_{max}=2$, and so on. Based on this idea, the solving process can be described as an induction process. For simplicity, we denote the expression $H_{max}=i$ as $H_{max}^{i}$. We first select all sensor nodes as cluster heads and solve the subproblem with $H_{max}^{0}$. Then, we solve other subproblems with $H_{max}^{i+1}=H_{max}^{i}+1$ based on the solution with $H_{max}^{i}$, where i=1,2,⋯,n.

Theorem 2 suggests that the RCP problem can be transformed as a TSP problem when $H_{max}=0$. Thus, it can be solved by classical TSP algorithms. However, when the maximum hops $H_{max}$ becomes larger, the RCP problem cannot be solved by TSP algorithms. For simplicity, but still effective, we update the solution with small maximum hops to achieve the solution with large maximum hops. Intuitively, we can shorten the travel route by reducing the number of cluster heads. Therefore, we first introduce a role change process to change some cluster heads to cluster members. Further more, in some cases, we can also shorten a travel route by increasing the number of cluster heads. Thus, we introduce another process to exchange cluster members with cluster heads. The two processes cannot guarantee that the updated solution is optimal, i.e., the process is not precise. Thus, the the proposed method is an imprecise induction method.

Figure 1 illustrates how the IIA algorithm works. When $H_{max}=0$, all sensor nodes must be cluster heads, and the travel route is $\rho_{H_{max}^{0}}=\langle y_{1}-x_{5}-x_{3}-x_{6}-x_{4}-x_{2}-x_{1}-y_{1}\rangle$. When $H_{max}=1$, the role of sensor node $x_{5}$ or sensor nodes $\left\{x_{1},x_{3}\right\}$ should be changed from cluster head to cluster member. If we set the sensor node $x_{5}$ as a cluster member, then the travel route is $\rho_{H_{max}^{1}}=\langle y_{1}-x_{3}-x_{6}-x_{4}-x_{2}-x_{1}-y_{1}\rangle$. However, $\rho_{H_{max}^{1}}$ is not the shortest one; we can exchange the role of sensor node $x_{3}$ and $x_{5}$ to achieve a shorter travel route, i.e., the sensor node $x_{3}$ is set as a cluster member, and the sensor node $x_{5}$ is set as a cluster head. Therefore, the travel route is updated as $\rho_{H_{max}^{1}}=\langle y_{1}-x_{5}-x_{6}-x_{4}-x_{2}-x_{1}-y_{1}\rangle$. The travel route is not the shortest one, either. If we set the sensor node $x_{1}$ as a cluster member, the travel route is $\rho_{H_{max}^{1}}=\langle y_{1}-x_{5}-x_{6}-x_{4}-x_{2}-y_{1}\rangle$. When $H_{max}=2$, the travel route cannot be updated further, so the travel route is $\rho_{H_{max}^{2}}=\langle y_{1}-x_{5}-x_{6}-x_{4}-x_{2}-y_{1}\rangle$.

### 4.2. Initial Travel Route

The initial travel route of the RCP problem is the optimal solution when $H_{max}=0$, which is a TSP problem from Theorem 2. When $H_{max}^{0}=0$, all sensor nodes are cluster heads, i.e., the decision variable U=1. Accordingly, the initial travel route problem can be derived from the RCP problem by setting U=1. The initial travel route problem is formulated as follows.

(14) $$\begin{array}{c} ITR:\min f\left(V\right)=\sum_{i=0}^{n}\sum_{j=0}^{n}v_{ij}d_{ij}\\ s.t. \end{array}$$

(15) $$\begin{array}{c} \sum_{i=0}^{n}v_{ij}=1,\left(j=0,1,\ldots ,n\right) \end{array}$$

(16) $$\begin{array}{c} \sum_{j=0}^{n}v_{ij}=1,\left(i=0,1,\ldots ,n\right) \end{array}$$

(17) $$\begin{array}{c} w_{i}-w_{j}+v_{ij}\left(n+1\right)=n,\left(i,j=0,1,\ldots ,n-1\right) \end{array}$$

(18) $$\begin{array}{c} v_{ij}\in \left\{0,1\right\} \end{array}$$

(19) $$\begin{array}{c} w_{i}\in \left\{1,2,\ldots ,n\right\} \end{array}$$

where the parameters and the constraints have the same meaning as the formulation of the RCP problem. The optimal solution of Equations (14)–(19) is denoted as ($U_{0}^{*}$, $V_{0}^{*}$), where the elements of $U_{0}^{*}$ are all one, and $V_{0}^{*}$ represents the travel route of the mobile sink.

### 4.3. Role Change from Cluster Head to Cluster Member

After a solution is achieved, we can get a shorter travel route by changing the role of a sensor node from cluster head to cluster member. Further more, when the solution ($U_{k}^{*}$, $V_{k}^{*})$ is achieved, we can solve the RCP problem of $H_{max}^{k+1}$ by setting some cluster heads in $U_{k}^{*}$ as a cluster member. If the sensor node $x_{p}$ changes its role from cluster head to cluster member, there are two matters: to remove the attached two edges and to connect two adjacent cluster heads by a link. The length of two attached edges can be calculated from the entering edge and leaving edge, which is denoted by Equation (20).

(20) $$\begin{array}{c} {\tilde{l}}_{x_{p}}=\sum_{j=1}^{n}\left(v_{pj}^{*}d_{pj}+v_{jp}^{*}d_{jp}\right) \end{array}$$

where ${\tilde{l}}_{x_{p}}$ is the total length of two attached edges of the sensor node $x_{p}$, the variables $v_{pj}$ and $v_{jp}$ denote whether the edges $\left(x_{p},x_{j}\right)$ and $\left(x_{j},x_{p}\right)$ are the attached edges and the parameters $d_{pj}$ and $d_{jp}$ indicate the length of the edges $\left(x_{p},x_{j}\right)$ and $\left(x_{j},x_{p}\right)$, respectively.

Whether an edge is set as a link is determined by the adjacent cluster heads of the sensor node $x_{p}$. We can achieve the link by transforming the decision variable matrix $V^{*}$, which is as follows. First, the $p^{\prime }th$ row of $V^{*}$ times all other rows as these rows’ value. Second, the $p^{\prime }th$ row of $V^{*}$ plus all other rows as these rows’ value. Third, the $p^{\prime }th$ column of $V^{*}$ times all other columns as these rows’ value.

According to these rules, the transformed decision variable $v_{ij}$ is calculated by Equation (21), and the length of the link is calculated by Equation (22).

(21) $$\begin{array}{c} {\hat{v}}_{ij}^{*}=v_{ip}^{*}\left(v_{pj}^{*}v_{ij}^{*}+v_{pj}^{*}\right) \end{array}$$

(22) $$\begin{array}{c} {\hat{l}}_{x_{p}}=\sum_{i}^{n}\sum_{j}^{n}v_{ip}^{*}d_{ip}\left(v_{pj}^{*}v_{ij}^{*}+v_{pj}^{*}\right) \end{array}$$

where ${\hat{v}}_{ij}^{*}$ denotes the transformed value of the decision variable $v_{ij}$ and ${\hat{l}}_{x_{p}}$ denotes the length of the added link.

In total, the decision variable $V^{*}$ should be transformed as Equation (23) when the role of the sensor node $x_{p}$ is changed from cluster head to cluster member.

(23) $$\begin{array}{c} {\hat{V}}^{*}\left(u_{p}\right)=\left\{\begin{array}{c} {\hat{v}}_{ij}^{*}=0,\;\left(i=p\;or\;j=p,u_{p}=1\right)\\ {\hat{v}}_{ij}^{*}=v_{ij}^{*}+v_{ip}^{*}\left(v_{pj}^{*}v_{ij}^{*}+v_{pj}^{*}\right),\;\left(others,u_{p}=1\right)\\ {\hat{v}}_{ij}^{*}=v_{ij}^{*},\;\left(u_{p}=0\right) \end{array}\right. \end{array}$$

where $u_{p}$ is the decision variable of the sensor node $x_{p}$ and ${\hat{V}}^{*}\left(u_{p}\right)$ is the transformed matrix of the decision variable $V^{*}$. Normally, $u_{p}=1$ if $x_{p}$ is a cluster head.

The reduced travel length when the role of the sensor node $x_{p}$ is changed from cluster head to cluster member is as follows.

(24) $$\begin{array}{c} \Delta l_{x_{p}}={\tilde{l}}_{x_{p}}-{\hat{l}}_{x_{p}} \end{array}$$

In the role change process, we want to achieve a shorter travel route, i.e., the reduced route length should be maximized. For a single sensor node, such as $x_{p}$, the following formulation decides whether the sensor node $x_{p}$ changes its cluster head role and maximizes the reduced route length.

(25) $$\begin{array}{c} H2M:\max f\left(u_{p}\right)=\left(u_{p}^{*}-u_{p}\right)(\sum_{j=1}^{n}\left(v_{pj}^{*}d_{pj}+v_{jp}^{*}d_{jp}\right)\\ -\sum_{i}^{n}\sum_{j}^{n}v_{ip}^{*}d_{ip}\left(v_{pj}^{*}v_{ij}^{*}+v_{pj}^{*}\right))\\ s.t. \end{array}$$

(26) $$\begin{array}{c} \left(1-u_{i}^{*}\right)\min_{j=1}^{n}u_{j}h_{ij}\leq H_{max},(i=1,2,\ldots ,n;\\ i\neq p;j\neq i) \end{array}$$

(27) $$\begin{array}{c} \left(1-u_{p}\right)\min_{j=1}^{n}u_{j}h_{pj}\leq H_{max} \end{array}$$

(28) $$\begin{array}{c} u_{p}\in \left\{0,1\right\} \end{array}$$

where $u_{p}$ is the decision variable that denotes whether the sensor node $x_{p}$ is selected as the cluster head. Equation (25) is the object function to maximize the reduced route length based on the solution ($U_{k}^{*}$, $V_{k}^{*})$. Equation (26) is the communication hop constraint, which restricts the maximum hops between cluster members and cluster heads, for all sensor nodes except the sensor node $x_{p}$. Equation (27) is also the communication hop constraint, which restricts the maximum hops among cluster members and the cluster head, especially for the sensor node $x_{p}$.

### 4.4. Role Exchange from Cluster Member to Cluster Head

This can further reduce the route length by exchanging the role between cluster member and cluster head. In mathematics, this kind of exchange can be mapped as vectors exchange on the matrix. If $x_{h}$ is a cluster head and $x_{c}$ is a cluster member, the matrix $V^{*}$ denotes a travel route and the matrix ${\hat{V}}^{*}$ is the exchanged travel route, then the exchanged travel route can be derived by Equations (29)–(32).

(29) $$\begin{array}{c} {\hat{v}}_{cj}^{*}=v_{hj}^{*},\left(j=1,2,\cdots ,n\right) \end{array}$$

(30) $$\begin{array}{c} {\hat{v}}_{ic}^{*}=v_{ih}^{*},\left(i=1,2,\cdots ,n\right) \end{array}$$

(31) $$\begin{array}{c} {\hat{v}}_{hj}^{*}=v_{cj}^{*},\left(j=1,2,\cdots ,n\right) \end{array}$$

(32) $$\begin{array}{c} {\hat{v}}_{ih}^{*}=v_{ic}^{*},\left(i=1,2,\cdots ,n\right) \end{array}$$

where $v_{ij}^{*}$ is the element of the matrix $V^{*}$ and ${\hat{v}}_{ij}^{*}$ is the element of the matrix ${\hat{V}}^{*}$.

Thus, the matrix of exchanged travel route ${\hat{V}}^{*}$ can be transformed by Equation (33).

(33) $$\begin{array}{c} {\hat{V}}^{*}\left(u_{h},u_{c}\right)=\left\{\begin{array}{c} {\hat{v}}_{ij}^{*}=v_{hj}^{*},\left(i=c,j=1,2,\cdots ,n\right)\\ {\hat{v}}_{ij}^{*}=v_{ih}^{*},\left(j=c,i=1,2,\cdots ,n\right)\\ {\hat{v}}_{ij}^{*}=v_{cj}^{*},\left(i=h,j=1,2,\cdots ,n\right)\\ {\hat{v}}_{ij}^{*}=v_{ic}^{*},\left(j=h,i=1,2,\cdots ,n\right)\\ {\hat{v}}_{ij}^{*}=v_{ij}^{*},\left(others\right) \end{array}\right. \end{array}$$

The reduced route length after role exchange between the cluster head $x_{h}$ and the cluster member $x_{c}$ can be calculated by Equation (34).

(34) $$\begin{array}{c} \Delta l_{x_{h}⟷x_{c}}=\sum_{i=1}^{n}v_{ih}^{*}d_{ih}+\sum_{j=1}^{n}v_{hj}^{*}d_{hj}\\ -\sum_{i=1}^{n}v_{ih}^{*}d_{ic}-\sum_{j=1}^{n}v_{hj}^{*}d_{cj} \end{array}$$

where $\Delta l_{x_{h}⟷x_{c}}$ is the reduced route length.

Similar to the role change process, the role exchange process is also to achieve a shorter travel route, i.e., the reduced route length should be maximized. For the cluster head $x_{h}$ and the cluster member $x_{c}$, the following formulation decides whether the sensor node $x_{h}$ should exchange with the sensor node $x_{c}$, so that the reduced route length is maximized.

(35) $$\begin{array}{c} M2H:\max f\left(u_{h},u_{c}\right)=\left(u_{h}^{*}-u_{h}\right)\left(u_{c}-u_{c}^{*}\right)(\sum_{i=1}^{n}v_{ih}^{*}d_{ih}\\ +\sum_{j=1}^{n}v_{hj}^{*}d_{hj}-\sum_{i=1}^{n}v_{ih}^{*}d_{ic}-\sum_{j=1}^{n}v_{hj}^{*}d_{cj})\\ s.t. \end{array}$$

(36) $$\begin{array}{c} \left(1-u_{i}^{*}\right)\min_{j=1}^{n}u_{j}h_{ij}\leq H_{max},(i=1,2,\ldots ,n;\\ i\neq h,i\neq c;j\neq i;) \end{array}$$

(37) $$\begin{array}{c} \left(1-u_{i}\right)\min_{j=1}^{n}u_{j}h_{ij}\leq H_{max},\left(i=\left\{h,c\right\}\right) \end{array}$$

(38) $$\begin{array}{c} u_{h},u_{c}\in \left\{0,1\right\} \end{array}$$

where $u_{h}$ and $u_{c}$ are decision variables that denote whether the cluster head $u_{h}$ should exchange with the cluster member $u_{c}$. Equation (35) is the object function to maximize the reduced route length based on the solution ($U_{k}^{*}$, $V_{k}^{*})$. Equations (36) and (37) are the constraints to restrict the maximum hops among cluster members and cluster heads, like Equations (26) and (27).

### 4.5. Details of the Imprecise Induction Algorithm

The IIA algorithm uses an iterative process to achieve the approximately optimal solution. At the beginning, the algorithm utilities a TSP algorithm to compute the solution of $H_{max}=0$. Then, the algorithm iteratively solves the problem with larger maximum hops by two sub-processes, such as with $H_{max}^{1}$, $H_{max}^{2}$, ⋯, $H_{max}^{k}$. The main process is as Algorithm 1, and the sub-processes are as Algorithms 2 and 3.
**Algorithm 1:** Imprecise Induction Algorithm (IIA). **Require:**  $H_{max}$: maximum hops among sensor nodes.  $\bar{X}$: the position of sensor nodes.  ${\bar{x}}_{0}$: the dock position of mobile sink.  $Md(\bar{X}⋃\left\{{\bar{x}}_{0}\right\})$: a function to calculate distances among sensor nodes.  $Mh(\bar{X}⋃\left\{{\bar{x}}_{0}\right\})$: a function to calculate minimum hop counts among sensor nodes by Floyd Algorithm.  $TSP_{itr}\left(md\right)$: a function to solute Equations (14)–(19) with distance parameter md.  $H2M(mh,U_{k-1}^{*},V_{k-1}^{*})$: a function to solve Algorithm 2.  $M2H(mh,U_{k-1}^{*},V_{k-1}^{*})$: a function to solve Algorithm 3. **Ensure:**  $U^{*}$: state vector of sensor node.  $V^{*}$: travel route of mobile sink.
1:$U_{0}^{*}\leftarrow 1$, k←02:$md\leftarrow Md(\bar{X}⋃\left\{{\bar{x}}_{0}\right\})$, $mh\leftarrow Mh(\bar{X}⋃\left\{{\bar{x}}_{0}\right\})$3:$V_{0}^{*}\leftarrow TSP_{itr}\left(md\right)$
4:**while** $k<H_{max}$ **do**5: k=k+1, δ=1;6: **while** δ>0 **do**7:  $\left(U_{k}^{♯},V_{k}^{♯}\right)\leftarrow H2M\left(mh,U_{k-1}^{*},V_{k-1}^{*}\right)$;8:  $\left(U_{k}^{*},V_{k}^{*}\right)\leftarrow M2H\left(mh,U_{k}^{♯},V_{k}^{♯}\right)$;9:  $\delta =f\left(U_{k}^{♯},V_{k}^{♯}\right)-f\left(U_{k}^{*},V_{k}^{*}\right)$;10: **end while**11:**end while**12:**return** $U_{k}^{*},V_{k}^{*}$

In Algorithm 1, Lines 1–3 assign the initial original value to key parameters and solve the RCP problem when $H_{max}=0$; Lines 4–11 solve the RCP problem iteratively when $H_{max}>0$. Line 5 applies the add-self operation to current maximum hops *k* and assigns the initial value to the temporary variable δ. Lines 6–10 will execute if there is a shorter travel route, i.e., δ>0. Lines 7–8 execute the role change process and role exchange process, respectively. Line 9 computes the object value gap between two update processes.

In Algorithm 2, Lines 2–4 calculate the maximum reduced route length for all sensor node; Line 5 sorts the maximum reduced route length by descending order; Lines 6–11 check the constraint to judge whether the sensor node can be a cluster member.

Like Algorithm 2, in Algorithm 3, Lines 2–4 calculate the maximum reduced route length for all sensor nodes; Line 5 sorts the maximum reduced route length by descending order; lines 6–11 check the constraint to judge whether the sensor node can be a cluster member.
**Algorithm 2:** Cluster Head to cluster Member (H2M) **Require:**  $\left(U_{k-1}^{*},V_{k-1}^{*}\right)$: the solution with $H_{max}=k-1$.  mh: the communication hops among sensor nodes.  $Maxf(u_{p},U_{k-1}^{*},V_{k-1}^{*})$: a function to calculate Equation (25) when the parameter $u_{p}==0$.  $Test(u_{p},mh)$: a function to test the parameter $u_{p}$ whether satisfies Equations (26) and (27).  Sort(L): a function to sort the collection *L* by descending order.  $V^{*}\left(u_{p}\right)$: a function to rebuild the travel route after $u_{p}\leftarrow 0$ by Equation (23). **Ensure:**  $U^{*}$: state vector of sensor node.  $V^{*}$: travel route of mobile sink. 1:L←∅2:**for** p=1 to *n* **do**3: $L\left(p\right)\leftarrow Maxf\left(u_{p},U_{k-1}^{*},V_{k-1}^{*}\right)$4:**end for**5:L←Sort(L)6:**for** i=1 to *n* **do**7: p←Index(L(i))8: **if** $U_{k}^{*}\left(p\right)==1$ and $Test(u_{p}\leftarrow 0,mh)$ **then**9:  $U_{k}^{*}\left(p\right)\leftarrow 0$, $V_{k}^{*}\leftarrow V^{*}\left(u_{p}\right)$10: **end if**11:**end for**12:**return** $U_{k}^{*},V_{k}^{*}$

**Algorithm 3:** Cluster Member to cluster Head (M2H) **Require:**  $\left(U_{k-1}^{*},V_{k-1}^{*}\right)$: the solution with $H_{max}=k-1$.  mh: the data transmission hops among sensor nodes.  $Maxf(u_{h},u_{c},U_{k-1}^{*},V_{k-1}^{*})$: a function to calculate Equation (35) with the parameter $u_{h}==0$ and $u_{c}==1$.  $Test(u_{h},u_{c},mh)$: a function to test the parameter $u_{h}$ and $u_{c}$ whether satisfies Equations (36) and (37).  Sort(L): a function to sort the collection *L* by descending order.  $V^{*}\left(u_{h},u_{c}\right)$: a function to rebuild the travel route after $u_{h}\leftarrow 0$ and $u_{c}\leftarrow 1$ by Equation (33). **Ensure:**  $U^{*}$: state vector of sensor node.  $V^{*}$: travel route of mobile sink. 1:L←∅
2:**for** p=1 to *n* **do**3: $L\left(p\right)\leftarrow Maxf\left(u_{p},u_{c},U_{k-1}^{*},V_{k-1}^{*}\right)$
4:**end for**5:L←Sort(L)
6:**for** i=1 to *n* **do**7: p←Index(L(i))8: **if** $Test(u_{h}\leftarrow 0,u_{c}\leftarrow 0,mh)$ **then**9:  $U_{k}^{*}\left(h\right)\leftarrow 0$,$U_{k}^{*}\left(c\right)\leftarrow 1$, $V_{k}^{*}\leftarrow V^{*}\left(u_{h},u_{c}\right)$10: **end if**11:**end for**12:**return** $U_{k}^{*},V_{k}^{*}$

## 5. Numerical Results

In this section, we provide numerical experiments to demonstrate the effectiveness of the proposed IIA algorithm and to compare the performance with the Shortest Path Tree-based Data-Gathering Algorithm (SPT-DGA) proposed in [14]. We first present the evaluation metrics and experimental settings.

### 5.1. Metrics and Settings

In the experiments, we define three metrics to evaluate the performance: route length, cluster head count and average hop count. Given the travel route matrix $V^{*}$ and the distance matrix Md, the route length is calculated by Equation (39). Given the state vector of sensor nodes $U^{*}$, the cluster head count is achieved by Equation (40). Given the state vector of sensor nodes $U^{*}$ and minimum hops matrix Mh, the average hop count is counted by Equation (41). The parameter settings are shown in Table 1.

(39) $$\begin{array}{c} f_{rl}\left(V^{*},M_{d}\right)=\sum_{i=1}^{n}\sum_{j=1}^{n}v_{ij}^{*}d_{ij} \end{array}$$

(40) $$\begin{array}{c} f_{chc}\left(U^{*}\right)=\sum_{i=1}^{n}u_{i} \end{array}$$

(41) $$\begin{array}{c} f_{ahc}\left(U^{*},M_{h}\right)=\frac{n+\sum_{i=1}^{n}\min_{>0}\left(M_{h}\left(i,1\cdots n\right)\cdot U^{*}\right)}{n} \end{array}$$

### 5.2. Experiment Results

**Figure 2:**
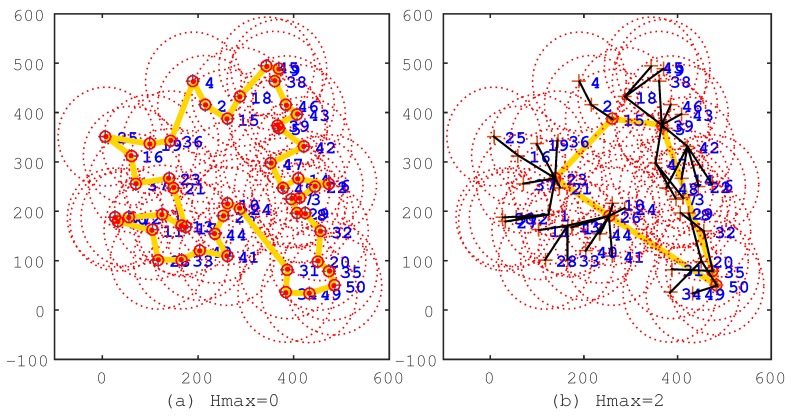
The solutions of the IIA algorithm under the conditions $H_{max}=0$ and $H_{max}=2$. (**a**) $H_{max}=0$; the route length is 1690.4 m; the cluster head count is 50; and the average hop count is one. (**b**) $H_{max}=2$; the route length is 1032.8 m; the cluster head count is 11; the average hop count is 1.75.

**Figure 3:**
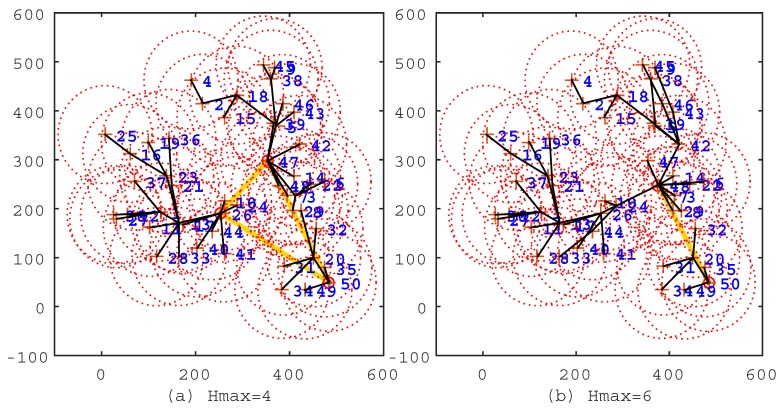
The solutions of the Imprecise Induction Algorithm (IIA) algorithm under the conditions $H_{max}=4$ and $H_{max}=6$. (**a**) $H_{max}=4$; the route length is 697.8 m; the cluster head count is three; and the average hop count is 3.57. (**b**) $H_{max}=6$; the route length is 452.6 m; the cluster head count is two; the average hop count is 5.3.

**Figure 4:**
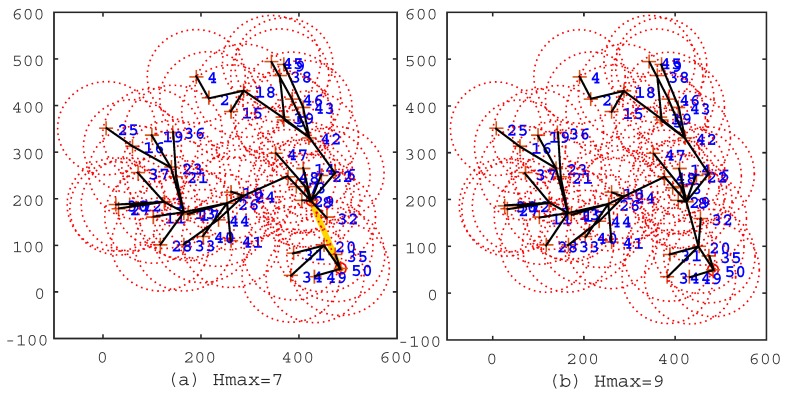
The solutions of the IIA algorithm under the conditions $H_{max}=7$ and $H_{max}=9$. (**a**) $H_{max}=7$; the route length is 315.1 m; the cluster head count is two; and the average hop count is 6.73. (**b**) $H_{max}=9$, the route length is 0 m; the cluster head count is one; the average hop count is 5.2.

To verify the effectiveness of the IIA algorithm, we first conduct experiments by deploying sensor nodes randomly on a plane with the parameters in Table 1. After that, we first use the IIA algorithm to select the cluster heads and to program a shorter travel route for the mobile sink. Then, by following [14], we use the shortest path algorithm (Floyd–Warshall algorithm) to establish the shortest data transmission path for sensor nodes, so that the average hop count could be smaller. From the minimum hops matrix Mh, we know that the maximum hops Hmax is within nine. We select typical solutions to demonstrate the processes that the IIA algorithm works effectively, as shown in Figure 2, Figure 3 and Figure 4.

In the figures, the black line segments denote communication links of sensor nodes; the circles denote cluster heads; and the khaki tour denotes the travel route of the mobile sink. In Figure 2a, $H_{max}=0$, which means that all sensor nodes are selected as cluster heads, and the travel route is a TSP tour. This travel route is the longest one among Figure 2, Figure 3 and Figure 4, but the data of sensor nodes can be transmitted to the mobile sink directly. In Figure 2b, $H_{max}=2$, which means that sensor nodes should transmit data within two hops. From this figure, we can see that most of the sensor nodes transmit data through two-hop links, and the travel route becomes definitely shorter. Figure 3, Figure 4 show the same situation as Figure 2b. It is worth mentioning that Figure 4b shows that the travel route cannot be further adjusted after $H_{max}=9$, because all of the sensor nodes can transmit their data to the mobile sink within nine hops. The experiments show that the IIA algorithm can automatically adjust cluster heads according to the parameter Hmax and plan a shorter travel route for the mobile sink.

**Figure 5:**
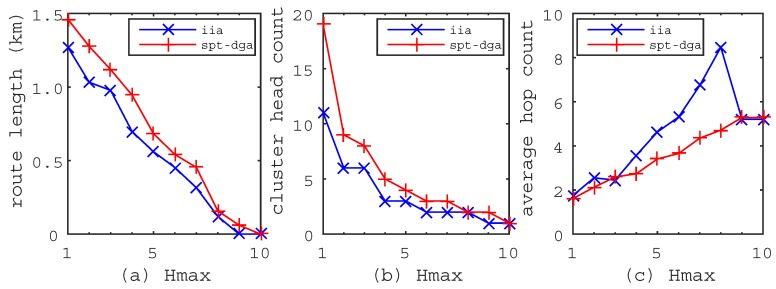
The metric variations when the maximum hops Hmax increases from 1–10. (**a**) Route length; the route length of the IIA algorithm is shorter than that of the Shortest Path Tree-based Data-Gathering Algorithm (SPT-DGA) algorithm. (**b**) Cluster head count; the cluster head count of the IIA algorithm is smaller than that of the SPT-DGA algorithm. (**c**) average hop count; the average hop count is higher than that of the SPT-DGA algorithm.

**Figure 6:**
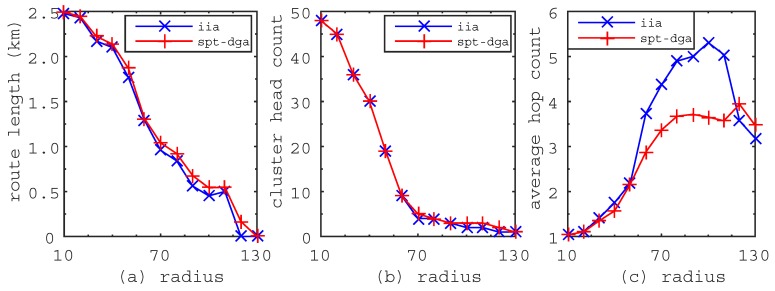
The metric variations when the communication radius increases from 10 m–130 m. (**a**) Route length; the route length decrease when the communication radius becomes large. (**b**) Cluster head count; the cluster head count decreases when the communication radius becomes large. (**c**) Average hop count; the average hop count increases when the communication becomes large.

We made performance comparisons between the IIA algorithm and the SPT-DGA algorithm proposed in [14]. The SPT-DGA algorithm includes three tasks. The first one is to construct the shortest path tree. The second one is to find cluster heads by the down to top approach, i.e., determine the cluster head from the leaf nodes to the root node. Additionally, the last one is to find the shortest tour visiting all cluster heads for the mobile sink. In the experiments, we use the Floyd–Warshall algorithm and the Dijkstra algorithm to achieve the shortest path tree for the IIA algorithm and the SPT-DGA algorithm, respectively. Additionally, we use the TSP solver provided by MATLAB to find the shortest travel route of the mobile sink for both of the algorithms. The performance comparison is based on the experiments of Figure 2, Figure 3 and Figure 4, and the results are shown in Figure 5, Figure 6.

Figure 5 shows the metric variations when the maximum hops Hmax increase from 1–10. From Figure 5a,b, we can see that the route length and the cluster head count will decrease when the maximum hops Hmax become large. However, the maximum hops Hmax are always bounded because we set the maximum hops to be the shortest path from the sensor nodes to the mobile sink, e.g., the maximum hops Hmax are less than 10 in Figure 2, Figure 3 and Figure 4. Figure 5a shows that the route length generated by the IIA algorithm is much shorter than that generated by the SPT-DGA algorithm. The route length of the IIA algorithm reaches 0 m when Hmax=9, but it is 62.7 m as obtained by the SPT-DGA algorithm. Figure 5b shows that the cluster head count obtained by the IIA algorithm is much smaller than that obtained by the SPT-DGA algorithm. Therefore, we conclude that the IIA algorithm has the characteristics of shorter route length, smaller cluster head count and faster convergence rate. From Figure 5c, we can see that the average hop count will increase when the maximum hops Hmax become small, but the maximum value is always less than Hmax. The average hop count of the IIA algorithm is much higher than that of the SPT-DGA algorithm, which leads us to conclude that a smaller cluster head count makes a higher average hop count in the RCP problem.

Figure 6 shows the metric variations when the communication radius increases from 10 m–130 m. From Figure 6a,b, we can see that the route length becomes shorter and the cluster head count becomes smaller when the communication radius of sensor nodes becomes larger. This is because the links of WSN will increase when the communication radius becomes large, which leads to more sensor nodes having the opportunity to become cluster heads. The newly-added cluster head creates the probability to make the route length shorter and the cluster head count smaller. Figure 6a shows that the route length generated by the IIA algorithm is always shorter than that generated by the SPT-DGA algorithm. Figure 6b shows that the cluster head count obtained by the IIA algorithm is smaller than that obtained by the SPT-DGA algorithm. Figure 6c shows that the average hop count of the IIA algorithm is much higher than that of the SPT-DGA algorithm. Figure 6a–c further confirm the characteristics of the IIA algorithm concluded from Figure 5. From the experiments, we can derive some laws as follows. First, the route length and the cluster head count decrease when the communication radius becomes large. Second, the average hop count increases when the communication radius becomes large. Third, the average hop count increases when the cluster head count becomes large. In general, the IIA algorithm can obtain a shorter route length, a smaller cluster head count and a higher average hop count than the SPT-DGA algorithm.

## 6. Conclusions

In this paper, we study the combination Route planning for mobile sink and Clustering Problem for static sensor nodes (RCP) in Wireless Sensor Networks with a Mobile Sink (WSN-MS). We formulate the RCP problem as an Integer Non-Linear Programming (INLP) problem. The objective is to shorten the travel route of the mobile sink with the constraints: the maximum hops constraint, the travel route constraint and the loop avoidance constraint. Since the RCP problem is hard to solve, we propose the Imprecise Induction Algorithm (IIA) to solve it. Extensive experiments show the characteristics of the RCP problem as follows. First, the route length and the cluster head count decrease when the communication radius becomes large. Second, the average hop count increases when the communication radius becomes large. Third, the average hop count increases when the cluster head count becomes small. From the experiments, we can see that the IIA algorithm could automatically adjust cluster heads according to the parameter Hmax and plan a shorter travel route for the mobile sink. Compared with the Shortest Path Tree-based Data-Gathering Algorithm (SPT-DGA), the IIA algorithm has the characteristics of shorter route length, smaller cluster head count and faster convergence rate.

## Figures and Tables

**Table 1:** Default parameters.

Parameter	Value	Comments
Ω	500 m × 500 m	The area in which are deployed sensor nodes.
$R_{max}$	100 m	The communication radius of sensor nodes.
*n*	50	The number of sensor nodes.
